# Infectious Pneumonia and Lung Ultrasound: A Review

**DOI:** 10.3390/jcm12041402

**Published:** 2023-02-10

**Authors:** Andrea Boccatonda, Giulio Cocco, Damiano D’Ardes, Andrea Delli Pizzi, Gianpaolo Vidili, Chiara De Molo, Susanna Vicari, Carla Serra, Francesco Cipollone, Cosima Schiavone, Maria Teresa Guagnano

**Affiliations:** 1 Internal Medicine, Bentivoglio Hospital, AUSL Bologna, 40010 Bologna, Italy; 2 Unit of Ultrasound in Internal Medicine, Department of Medicine and Science of Aging, G. D’Annunzio University, 66100 Chieti, Italy; 3 Internal Medicine, Department of Medicine and Science of Aging, G. D’Annunzio University, 66100 Chieti, Italy; 4 Unit of Radiology, “Santissima Annunziata” Hospital, 66100 Chieti, Italy; 5 Department of Medicine, Surgery and Pharmacy, University of Sassari, 07100 Sassari, Italy; 6 Interventional, Diagnostic and Therapeutic Ultrasound Unit, IRCCS, Azienda Ospedaliero-Universitaria di Bologna, 40010 Bologna, Italy

**Keywords:** pneumonia, lung, ultrasound, COVID-19, heart failure, cancer, consolidation

## Abstract

The application of thoracic ultrasound examination has not long been developed because ultrasound’s interaction with the lung does not generate an anatomical image but an artifactual one. Subsequently, the evaluation of pulmonary artifacts and their correlation to specific diseases allowed the development of ultrasound semantics. Currently, pneumonia still represents one of the main causes of hospitalization and mortality. Several studies in the literature have demonstrated the ultrasound features of pneumonia. Although ultrasound cannot be considered the diagnostic gold standard for the study of all lung diseases, it has experienced an extraordinary development and growth of interest due to the SARS-CoV-2 pandemic. This review aims to provide essential information on the application of lung ultrasound to the study of infectious pneumonia and to discuss the differential diagnosis.

## 1. Introduction

In the past, the lung did not represent one of the fields of application for ultrasound due to the presence of tissues with a high difference in acoustic impedance, hindering the propagation of ultrasounds (air in the alveoli vs. the osteomuscular tissues of the rib cage) [1]. Air reflects up to 99% of the ultrasounds, thus generating shadow cones and reverberation artifacts; the bone reflects the ultrasound almost completely, also generating posterior shadow cones. Those structures hinder the visualization of the lung. Acoustic artifacts generated by the interaction of ultrasound with air appear different depending on the air content and the homogeneity of its distribution. Therefore, ultrasound evaluation of the lung is now based on the study of artifacts [2,3].

The technique was initially applied mainly in the field of emergency medicine and intensive care [2,4,5,6], but over the last years, its use has extended to the internal medicine and specialist fields [7]. Infectious pneumonia has always been among the most relevant diseases in the medical field due to its high number of complications and mortality [8,9,10,11].

Therefore, a large number of studies on lung ultrasound have been dedicated to the evaluation of pneumonia and to the definition of specific signs that would allow a differential diagnosis [8,9,10,11]. In particular, lung ultrasound has found exponentially growing use and interest with the advent of the SARS-CoV-2 pandemic [12,13,14,15].

This review aims to provide essential information on the application of lung ultrasound to the study of infectious pneumonia and to discuss the differential diagnosis.

## 2. Basic Lung Ultrasound

Lung ultrasound can be performed by a normal basic device, simply through B-mode evaluation, even without color-doppler functions. Different transducers can be used, such as a low-frequency convex probe (from 1 to 5 MHz) that allows viewing in-depth but with a low lateral resolution of the image, a high-frequency linear probe (7–18 MHz) characterized by poor in-depth penetration but allows observing the superficial structures (pleural line) with a high resolution. The sector or cardiological probe (4–2 MHz) can also be used, thus presenting the advantage of the simplest positioning in the intercostal space. It is advisable to eliminate the use of harmonics and image self-enhancement programs on ultrasound machines to favor the visualization of artifacts; it is also advisable to set the total image gain to a low degree and set the focus at the level of the pleural line.

The patient can be examined in any position, most frequently supine or sitting, depending on their clinical condition. Bed-ridden and intensive care unit (ICU) patients can often be examined in the lateral position. By asking the patient to raise his arm above the head or to place it on the opposite shoulder, the intercostal spaces are more expanded and accessible.

The scan planes are divided into posterior to the thorax with intercostal, longitudinal, transverse, and paravertebral scans; anteriorly, supra and parasternal, subxiphoid, and supraclavicular scans are used. The lung scans vertically follow the parasternal, hemiclavicular, axillary, and paravertebral anatomical lines, and horizontally, the intercostal spaces produce longitudinal coronal sections.

Some segments of the thorax remain invisible to ultrasound, such as the retroscapular and retrosternal regions, a portion of the mediastinal parietal pleura, and the costovertebral recess area.

There is no consensus on the number of scans to be performed and on which lung fields [13,14,16]. In an emergency setting, a quick examination is mostly employed, characterized by a few bilateral scans, generally 6 for each hemithorax (2 anterior, 2 lateral, and 2 posterior) [17]. In other settings, multiple scans can be performed to improve diagnostic accuracy, particularly for signs of focal pulmonary changes [18]. Regarding the evaluation of a patient with suspected pneumonia, the site of the posterolateral and/or pleural alveolar syndrome (PLAPS) is usually indicated as the area of choice [2,3]. Undoubtedly, the localization of the specific infection depends on its pathogenetic mechanism (lobar vs. viral interstitial bacterial pneumonia) [6,8,11].

The use of a common scheme and a common ultrasound semiotic allows a serial follow-up over time, evaluating the changes in the sonographic characteristics of the lung fields examined [15,19].

## 3. Lung Ultrasound Evaluation

**Figure 1:**
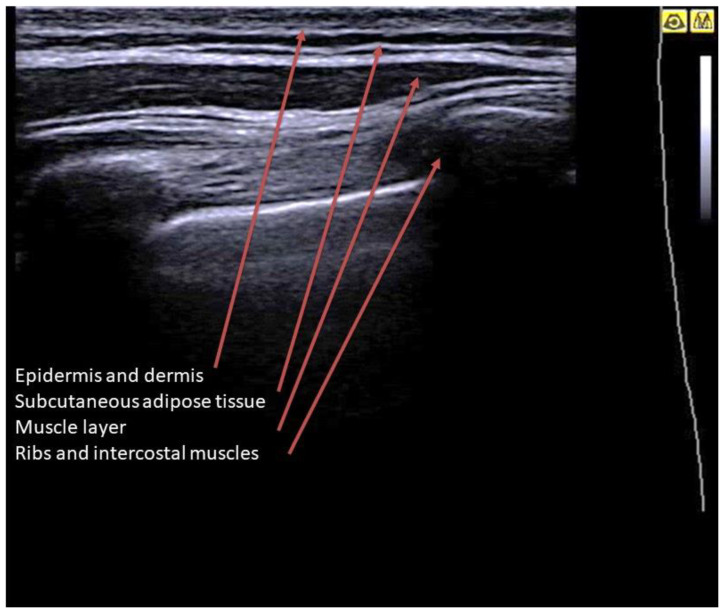
Longitudinal scan on a B-mode image of a healthy lung. Image showing from the surface to the depth: a homogeneous hyperechoic layer given by the skin (epidermis and dermis); a layer with mixed echogenicity (anechoic with hyperechoic striae) which represents the subcutaneous adipose tissue; the muscular layer, with a predominantly hypoechoic appearance; deeper layer: the ribs and intercostal muscles.

By placing the probe on the skin in longitudinal scans, it is possible to visualize the superficial layers of the wall (Figure 1). The ribs completely reflect the ultrasound and can be studied only in the cortical portion, which appears as a hyperechoic line overlying a shadow cone. The rib section can be variable depending on the scan: curved cortex and rounded section in posterior scans, flat cortex and rectangular section in lateral scans. The costal cartilages have a lower impedance, and although they determine a shadow cone, they can reveal the underlying structures (depending on age, they may contain hyperechoic calcifications). The ribs and sternum are also observable along their major axis. The intercostal muscles are visible as horizontal hypoechoic bundles between the ribs [20].

## 4. Lung Ultrasound on Physiological Lung

**Figure 2:**
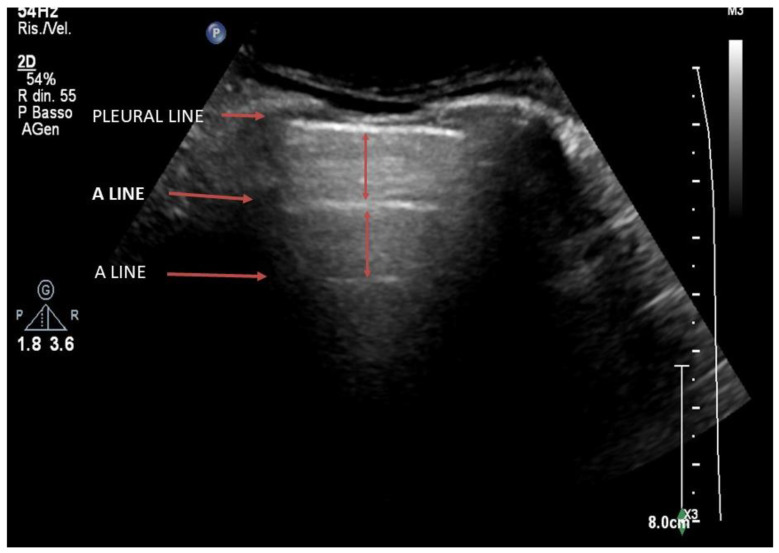
Longitudinal scan on a B-mode image of a healthy lung. Below the ribs, there is a longitudinal hyperechoic line which is called the pleural line. In-depth, horizontal reverberation artifacts are evident, defined as A-lines.

Below the structures of the rib cage, the presence of the lung parenchyma containing air will totally reflect the ultrasound due to the high difference in acoustic impedance (Figure 2). On ultrasound imaging, the area between the soft tissue interface and the ventilated lung will appear as a linear hyperechoic structure with a horizontal movement called “sliding” that is synchronous with the ventilation [21] (Supplementary Materials Video S1). This hyperechoic line is commonly identified as the “pleural line”, even if it does not correspond anatomically to the visceral or parietal pleura, which would not be visible by ultrasound.

The complete reflection of ultrasound on the pleural line determines the formation of artifacts which, on the ultrasound image, represent the lung parenchyma. The ultrasound imaging of a normally ventilated lung is represented by horizontal artifacts immediately below the thoracic cage due to the considerable difference in acoustic impedance between soft tissue and ventilated lung (reverberation artifacts or A-lines). Observing the lung in the posterior longitudinal scan, at the base of the lung, we can see a point where the pleural line and the underlying artifacts are interrupted to shift into the abdomen. At this point, the base of the lung moves like a “curtain” during the respiratory acts, generating the “curtain sign”, which identifies the lung base (Supplementary Materials Video S2).

It is necessary to specify that a normal pattern with A-lines is not always physiological; indeed, pulmonary diseases affecting vessels, such as pulmonary embolism, and pathologies that primarily affect the bronchial branches, such as chronic obstructive pulmonary disease (COPD), present with a “normal” A-line ultrasound feature. Moreover, A-lines can also be detected in the case of pneumothorax, but in this case, the pleural sliding is abolished.

## 5. From the B-Lines to the Pathological Lung

### 5.1. Interstitial Syndrome

**Figure 3:**
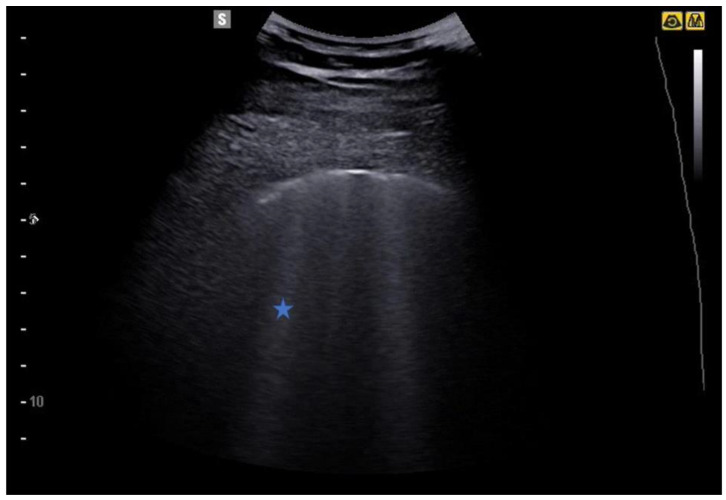
B-lines (star) are laser-like vertical hyperechoic reverberation artifacts arising from the pleural line extending to the bottom of the screen without fading and moving synchronously with lung sliding.

The subpleural air interface also allows the detection of vertical artifacts. Laser-like vertical hyperechoic reverberation artifacts arising from the pleural line are called B-lines (also called “comet tails”), extending to the bottom of the screen without fading and moving synchronously with lung sliding (Figure 3) [22].

**Figure 4:**
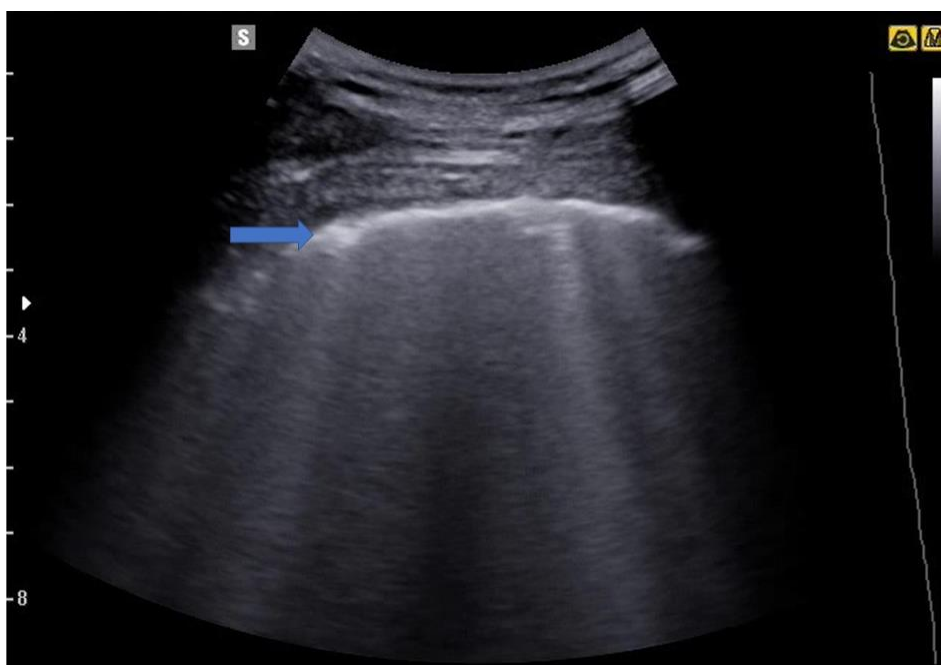
Image of a patient suffering from mild COVID-19 pneumonia. The blue arrow indicates an irregular pleural line, an early sign of covid-19 pneumonia. Some B lines are also evident in the image.

**Figure 5:**
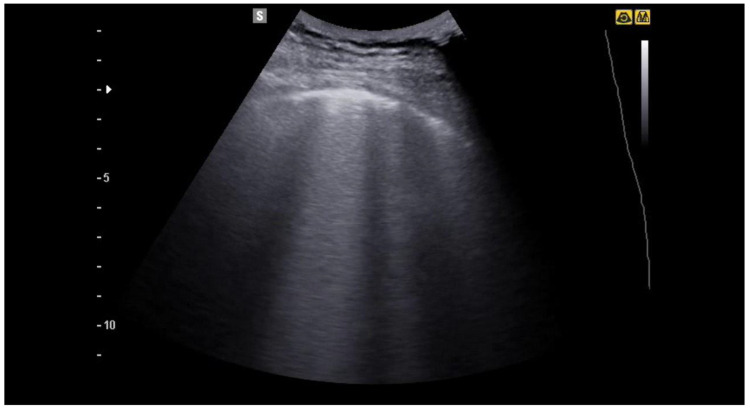
In the image the irregularity of the pleural line and some B lines are evident, which in the middle part of the image merge to form a larger artefact.

**Figure 6:**
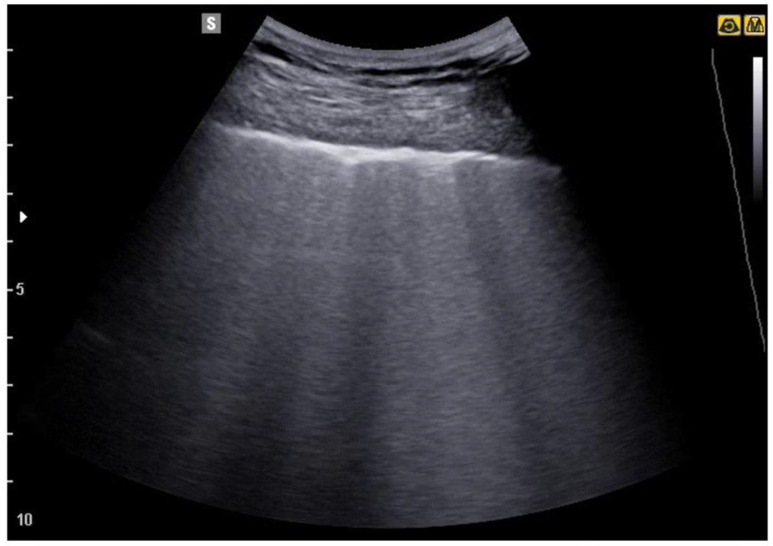
The image shows a more severe picture of interstitial disease than the previous ones. Multiple areas of confluence of lines B are highlighted.

**Figure 7:**
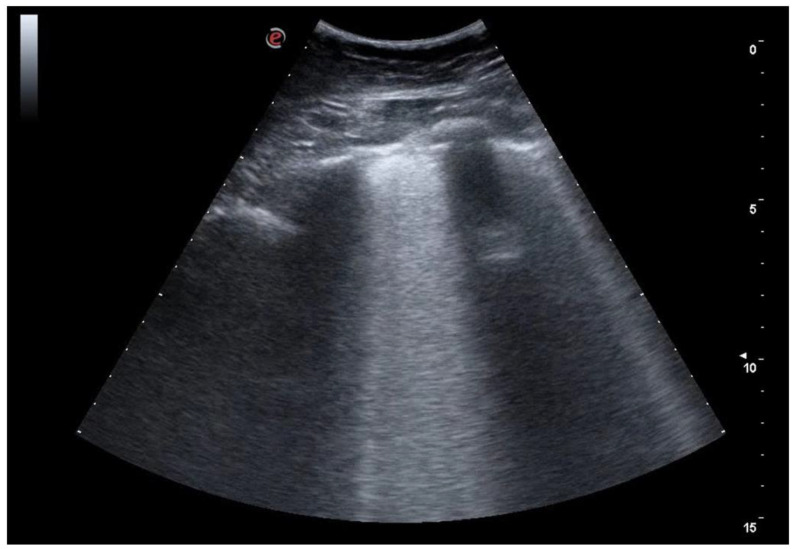
Severe interstitial disease. Many B lines are evident which merge to occupy the entire pulmonary field, to form the so-called "white lung".

Although the pathophysiological basis of B-lines is not known, they are a sign of lung interstitial syndrome. It should be noted that fewer than 3 lines per lung field examined is considered normal, especially in elderly subjects. When the B-lines occupy more than 50% of the lung field examined, until it is full, as if to form a single front of hyperechoic lines, it takes the name of “white lung” [22,23] (Figure 4, Figure 5, Figure 6 and Figure 7).

Nowadays, the most cited hypothesis to explain the genesis of B-lines is based on the concept that the pulmonary cortex (the most peripheral lung area near the pleura) is like a microsponge, consisting of air spaces (peripheral alveoli) surrounded by interstitium [24,25,26]. When the ultrasound interacts with that physiological interface, the normal A-line pattern previously described is generated; on the other hand, a progressive accumulation of fluids induced by a pathological state (exudate or transudate) at the interstitial level induces a concomitant reduction in the amount of alveolar air [24,25,26]. The progressive loss of air changes the interface with the pleura, thus disrupting the layer of microbubbles; that change allows a greater amount of ultrasound to penetrate deeply and be partially reflected in an irregular manner, thus generating hyperechoic reverberation artifacts [24,25,26].

The detection of multiple diffuse bilateral B-lines is suggestive of interstitial syndrome (pulmonary edema of various causes, interstitial pneumonia or pneumonitis, diffuse parenchymal lung disease). Focal multiple B-lines (>3) can be suggestive of focal interstitial syndrome (pneumonia, contusion, pulmonary infarction, lung cancer, and pleural diseases) [24,25,26]. Many works have investigated the role of B-lines in the diagnosis and monitoring of patients with cardiogenic pulmonary edema. In those patients, the B-lines are bilateral with a typical base–apex gradient [25].

In pulmonary fibrosis, B-lines are bilateral, diffuse, and characterized by a nonhomogeneous distribution. Other ultrasound signs suggestive of pulmonary fibrosis include irregular pleural lines and small subpleural consolidations [27,28,29,30,31,32,33].

On lung ultrasound, acute respiratory distress syndrome (ARDS) is also characterized by a nonhomogeneous distribution of B-lines and irregular pleural lines; the presence of anterior subpleural consolidations, the reduction of lung sliding, and the detection of “spared areas” of normal lung parenchyma are other specific features [34,35,36].

Intriguingly, many of those ultrasound features typical of pulmonary fibrosis and ARDS have been found in the new COVID-19 pneumonia.

### 5.2. Lung Consolidations

With the further loss of alveolar air content and the simultaneous increase in fluids, the absence of air in the alveoli induces the appearance of a consolidative pattern. When the pulmonary consolidation area reaches the pleural line, and there is no interposition of normal parenchyma between the probe and the pathological focus, ultrasound can represent it as a “real” picture. Consolidation appears as a diffusely hypoechoic and compact area, such as a liver (hepatization), and in the context of which anatomical features such as vascularization and branches of the bronchial tree can be visible. In the case of maintained airway patency, hyperechoic spots may also be visible within the lung parenchyma due to the air present in the bronchial tree, which generates hyperechoic points and/or linear images called air bronchograms [37].

Areas of consolidation can be an expression of inflammatory diseases such as pneumonia but also of neoplastic, macronodular granulomatous, alveolar, infarct, and atelectasis diseases. The analysis of the margins, the number, the eco-structural aspects, and the characteristics of the air bronchograms was proposed to differentiate the various types of lung consolidation [38].

## 6. Pneumonia

Pneumonia is the third most common disease for hospital admission, accounting for 544,000/year hospitalizations from the emergency department [39]. Moreover, pneumonia is the leading cause of sepsis and death from infection [39]. Pneumonia can be divided into four categories: CAP, healthcare-associated pneumonia (HCAP), hospital-acquired pneumonia, and ventilator-associated pneumonia [40].

Only 38% of patients hospitalized for pneumonia had a pathogen identified, and most were viral, according to a population-based surveillance study [41]. A bacterial pathogen was detected in only 14% of cases [41]. The most common pathogen was rhinovirus, followed by the influenza virus, then Streptococcus pneumoniae. Mycoplasma pneumoniae and Staphylococcus aureus were the second and third most common bacterial pathogens, respectively. Other bacterial species are Legionella pneumophila, Haemophilus influenzae, Chlamydophila pneumoniae, and Moraxella catarrhalis [39,41]. Other viral causes include human metapneumovirus, parainfluenza virus, respiratory syncytial virus, coronavirus, and adenovirus [39]. Influenza can be the most common cause of community-acquired pneumonia (CAP) requiring hospitalization during peak influenza season, and it can be complicated by secondary bacterial infection. Fungal pneumonia is generally detected in patients affected by immune deficits.

Pneumocystic jiroveci, Aspergillus, Candida albicans, and Cryptococcus neoformans are the most frequently detected opportunistic fungal pneumonia in patients with acquired immune deficiency syndrome (AIDS) and solid organ transplants.

At the beginning of 2020, the SARS-CoV-2 pandemic changed pneumonia epidemiology, being the most frequent and relevant pneumonia cause requiring hospitalization [42].

Anatomically, pneumonia are divided into lobar, with the consolidation of one or more lobes; bronchopneumonia (staphylococcus), the inflammatory process is confined by the interlobular septa and manifests itself with parenchymal or patchy thickening; interstitial pneumonia (viruses and mycoplasma) in which the alveolar exudate, if present, is not so marked; and mixed pneumonia [11,43,44].

Histopathological features of pneumonia can be differentiated on the basis of radiological and ultrasound signs [11,44] (Table 1). Lobar pneumonia appears on ultrasound as lobar consolidation, interstitial pneumonia is detected as B-lines and white lung areas, with consolidation in most serious cases, and mixed-bronchopneumonia appears as small subpleural consolidation with surrounding B-lines.

**Figure 8:**
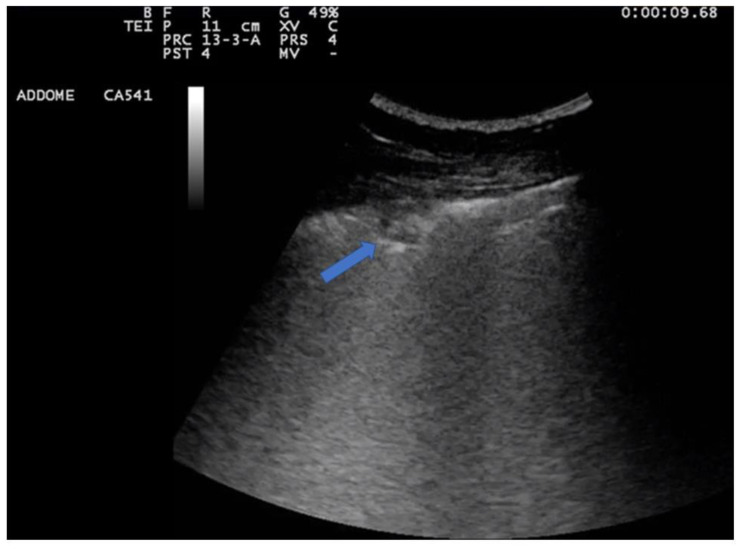
The image shows an interruption of the pleural line with a subpleural consolidation (blue arrow) with the presence of some bronchiolograms inside. That finding is typical for bronchopneumonia in a patient with fever and is clinically suggestive of ongoing infection.

**Figure 9:**
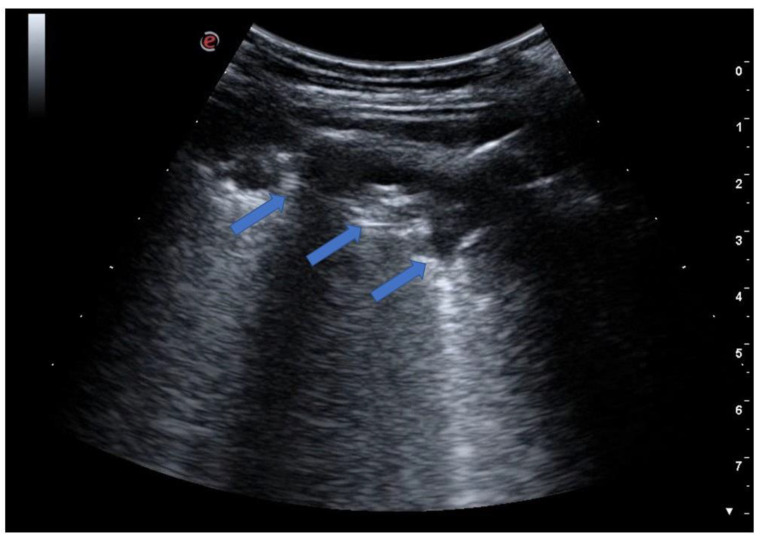
The image shows a large subpleural consolidation (blue arrows) extending over approximately two intercostal spaces with internal bronchograms. The image is typical for a picture of pneumonia with initial lobar involvement.

Pneumonia can be seen on ultrasound—provided that it reaches the pleural line—as hypoechoic areas (Figure 8) of variable size with sonographic characteristics similar to hepatic/splenic parenchyma (so-called liver-like tissue sign); when present, this ultrasound sign is of high sensitivity (90%) and specificity (98%) [45]. Another diagnostic ultrasound sign is the so-called “shred sign”, showing a blurred and fragmented margin separating the consolidation area from the aerated parenchyma (Figure 9).

The involvement of the pleura by the consolidation induces an interruption of the pleural line, and the sliding is significantly reduced or absent at this point.

**Figure 10:**
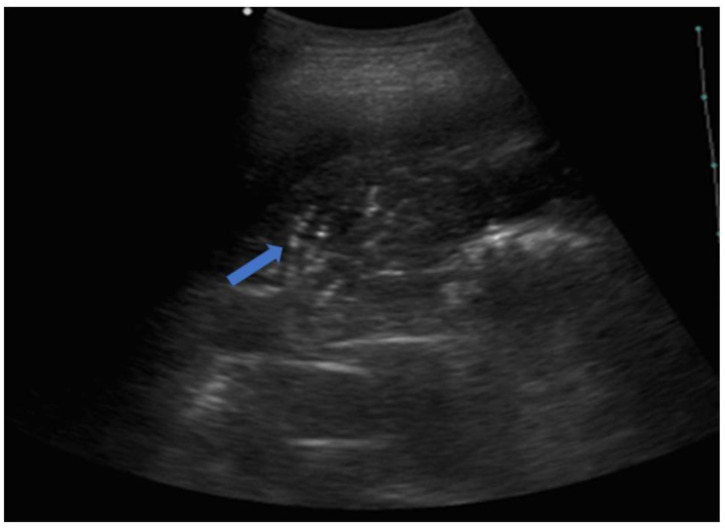
The image shows a complete lobar consolidation with air bronchograms (blue arrow) and minimal parapneumonic pleural effusion (lobar pneumonia). The air interface is completely lost, and the lung lobe is represented as a parenchymatous organ similar to the hepatic parenchyma (lung hepatization).

In adults, infectious lung consolidations reach the pleura in 98.5% of cases [46]. Another typical feature is represented by the presence of air bronchograms or the presence of hyperechoic or linear punctate spots within the lung consolidation [47] (Figure 10). The air bronchogram is seen in over 90% of patients with pneumonia [10,47]. Even more specific is the presence of dynamic air bronchograms or branched echogenic structures within the area of alveolar consolidation with centrifugal movements with inspiration [48]. The presence of dynamic air bronchograms has a high positive predictive value in the diagnosis of pneumonia and allows the differential ultrasound diagnosis with obstructive atelectasis, which in many cases shows static air bronchograms [48].

Furthermore, the fluid bronchogram is identified by anechoic/hypoechoic branched tubular structures in relation to the bronchial tree [49]. Bronchial obstructions (caused by mucus plaques and neoplasia) should be considered in the case of a persistent fluid bronchogram [50] and should be confirmed/treated with appropriate intervention (e.g., bronchoscopy) [37].

The presence within lung parenchyma of tubular structures with echogenic walls and no color-doppler signal represent fluid-filled bronchi (fluid bronchograms), typical of post-obstructive pneumonia. It should also be noted that in the course of pneumonia, ultrasound shows a consensual pleural effusion in 55% of cases, significantly higher than the standard x-ray examination, where the percentage reaches 25% [11].

In the later stages, the echo pattern becomes more dense and inhomogeneous. The reappearance of echogenic images in the bronchi and alveoli can be seen as a sign of healing and as a sign of re-ventilation of the lung parenchyma. Reverberation artifacts are the typical ultrasound signs visible at this stage.

In a multicenter study on 226 patients, ultrasound showed a sensitivity of 93.4% and a specificity of 97.7% for the diagnosis of pneumonia [51]. Liver-like consolidation, the presence of air bronchograms, and indistinct margins represent the most predictive characteristics of pneumonia. Similar results are reported in the meta-analysis by Chavez, with a cumulative sensitivity of 94% and a specificity of 96% [52]. One study performed on 79 pediatric patients demonstrated that lung ultrasound displays a higher sensitivity than chest X-ray [44]. In two studies performed in adults, lung ultrasound was superior to the standard radiological examination [6,53].

Viral pneumonia is characterized by the presence of small subpleural consolidations less than 5 mm, single and focal with multiple and diffuse B-line artifacts (white lung sign), small pleural effusions, and pleural line abnormalities (thickened > 2 mm) [54,55].

Abnormalities in viral pneumonia most frequently occur within lower lung fields, over the posterior and lateral chest surface [55].

Infectious pneumonia displays relevant vascularization, especially in the acute phase, through the pulmonary artery [45,52]. A significant arterial vascularization with a high resistance index (RI > 0.8) has typically been shown within the lesion [11,45,51,52]. However, that finding can also be evidenced in atelectasis. Otherwise, neoplastic lesions are characterized by predominantly low-resistance bronchial vascularity with irregularly distributed vessels [11,51,52]. Pulmonary infarcts, on the other hand, are characterized by absent or poor peripheral vascularization [51,52].

Those features are better detected by CEUS. Pneumonia is characterized by an early and homogeneous enhancement after 5–7 s (pulmonary artery flow); a delay in the acquisition of contrast can be observed in lobar pneumonia due to vasoconstriction [56,57,58]. Neoplastic and metastatic lesions present mainly bronchial, variable, and inhomogeneous vascularization, thus displaying a delay in enhancement of about 18–20 s [56,57,58]. Necrotic and colliquative lesions do not intake contrast, as well as pulmonary infarcts [56].

Recent studies evaluated the role of elastosonography in differentiating benign vs. malignant consolidation. Our group reported a preliminary report on patients with subpleural consolidations that performed elastosonography evaluation; malignant lesions were characterized by higher values of tissue stiffness than benign ones [59].

## 7. Lung Abscess

Ultrasound evaluation can detect early pneumonia complications, such as the formation of abscesses. Abscesses are quite common, particularly after a staphylococcus infection [60]. The ultrasound image of abscesses is given by focal consolidations with irregular and hypoechoic edges. Later in the course of the disease, these areas are progressively isolated, and the surrounding lung tissue has a hyperechoic border. Color-doppler function reveals marked vascularization, and contrast-enhanced ultrasound (CEUS) shows early and intense contrast enhancement [9,61,62]. CEUS is useful for the differential diagnosis of abscesses by microvascular analysis: abscesses are not vascularized, while neo-angiogenesis is generally present in neoplastic lesions. In case of resistance to antibiotic therapy, CEUS-guided biopsy can be used to drain the abscess and provide samples for microbiological examinations [63].

## 8. Pulmonary Tuberculosis

Pulmonary tuberculosis can present with a variety of mediastinal, pleural, and pulmonary changes, such as pleural effusion and rupture of the visceral pleura with subpleural consolidations and the formation of cavities or abscesses [64]. Tuberculosis lesions are often irregularly delineated with a homogeneously hypoechoic texture [64]. Miliary tuberculosis is characterized by ultrasound by multiple small hypoechoic subpleural nodules (<5 mm) [65]. Despite this evidence, CT remains the standard gold method in the evaluation of suspected pulmonary tuberculosis.

## 9. Lung Ultrasound in COVID-19 Pneumonia

The typical ultrasound feature of early interstitial pneumonia is represented by B-lines arising from an irregular pleural line, frequently related to small subpleural consolidations [66]; there is often no homogeneity between the different lines, even in the same lung field [13,15,67,68]. This is probably due to a severe change in the subpleural pulmonary interstitium. Therefore, the features of COVID-19 pneumonia on lung ultrasound have been described as “a storm of B-lines” [12,13,69].

An increasing number of B-lines correlates with a more significant pathological change in the lung [6,68]. In COVID-19 patients, preliminary data have shown that there is a correspondence between white lung on ultrasound and ground glass appearance on high-resolution computed tomography HRCT [15,67,70]. When the pathological process leads to a total loss of air content, the lung parenchyma consolidates [15,67,70]. Therefore, subpleural consolidations are found in the lung fields most severely damaged by the infectious process.

In COVID-19 patients with severe respiratory failure, areas of atelectasis due to poor ventilation and muscle overload can also be highlighted, especially on basal fields [14,15,68]. Moreover, the possibility of bacterial superinfection should always be considered, which can manifest itself with extensive lung consolidation and dynamic air bronchogram [14,15,68].

A less frequent finding is a pleural effusion, which is detected in only 4.7% of patients, according to data from work by Lomoro [71] and in 10% of patients in another report [67]. According to recent evidence, the finding of pleural effusion in COVID-19 pneumonia seems to be related to more severe disease. The lack of pleural sliding and the finding of lung point can be detected in the case of pneumothorax due to a very serious alteration of the lung parenchyma or mechanical ventilation barotrauma [15,68,72].

In COVID-19 pneumonia, the changes are mainly peripheral, bilateral, and not homogeneously distributed (patchwork pattern), with sparing areas [13,14,15,67,68,73]. In particular, the finding of pathological lung areas (white lung) in mid-apical sites, but with basal sparing, represents a highly suspicious ultrasound picture for COVID-19 pneumonia [14,74]. Furthermore, recent data have reinforced the concept that SARS-CoV-19 pneumonia exhibits the bilateral distribution of multiform clusters of B-lines, alternating with sparing areas, suggesting ultrasound probability models for COVID-19 pneumonia based on ultrasound signs [13,14,66,68].

## 10. Fields of Application of Lung Ultrasound

According to the literature, it is evident that lung ultrasound is certainly not inferior and, in some studies, even superior to the standard X-ray examination of the chest [46,75]; therefore, it can represent a relevant alternative as well as an integration to radiological examination both in emergency and ordinary hospitalizations [11,44]. Lung ultrasound is certainly indicated in those patients in which it is preferable to avoid exposure to ionizing radiation, such as in pregnancy and in children [11,44].

Frail, elderly patients are often admitted to COVID-19 wards and characterized by disabilities and comorbidities and with multiple therapies. In those patients, it is often not possible to perform a chest X-ray examination in two projections in optimal conditions due to hypokinesia, tremors, as well as difficulty in maintaining an upright position, and inspiratory apnea.

Lung ultrasound can detect more pneumonia (12–25%) than X-rays, as confirmed by chest CT [6,44]. Lung ultrasound displays a sensitivity of 93.4% and specificity of 97.7% for the diagnosis of community-acquired pneumonia [6,44].

However, about 8% of pneumonic lesions are undetectable by lung ultrasound; therefore, a non-diagnostic lung ultrasound does not exclude the diagnosis of pneumonia [51].

Lung ultrasound is a useful tool for the follow-up of patients with pneumonia and for monitoring the effectiveness of the therapy and the healing process [76,77]; in particular, the reduction of consolidations, and the reappearance of the signs of aeration (bronchograms), seems to be a good strategy [11,77]. Therefore, lung ultrasound can be a screening or first-level method in the diagnosis of pneumonia to be integrated into the clinical context, especially in the pediatric field and in all situations in which it is not possible to perform a radiological method such as chest X-ray or CT scan. Chest CT remains the gold-standard method, while chest radiography can find a role as a completion method when there is a high clinical suspicion despite a negative ultrasound examination.

## 11. Conclusions

Lung ultrasound is a method based on the study of artifacts. Different lung diseases are related to different artefactual aspects. The ultrasound appearance of pneumonia depends on its etiology and pathogenesis. Generally, viral etiologies and those from atypical bacteria are characterized by a B-line pattern, at least in the initial phase. Bacterial etiologies are characterized by the formation of lung consolidations up to the striking picture of streptococcal lobar pneumonia. Viral pneumonia due to SARS-CoV-2 infection is characterized by multiple B-lines with nonhomogeneous distribution with sparing areas during its initial phase; in the most severe forms, consolidations may appear. Ultrasound can be useful in monitoring clinical evolution, verifying complications, and as a guide for diagnostic–interventional procedures.

## Figures and Tables

**Table 1:** Differential diagnosis and ultrasound features of pneumonia.

	Features	Complications
Streptococcus pneumoniae	Lobar consolidation	Pleural effusion
Staphilococcus aureus	Multifocal consolidations	Abscess-empyema
Klebsiella pneumoniae	Upper lobes consolidations	Abscess
Pseudomonas aeruginosa	Multifocal consolidations	Abscess
Haemophilus influenzae, legionella pneumophila, moraxella catarralis, chlamidia pneumoniae, mycoplasma pneumoniae	Multifocal B-lines	Consolidation, pleural effusion
Anaerobes	Upper lobes consolidations	Abscess

## Supplementary Materials

The following supporting information can be downloaded at: https://www.mdpi.com/article/10.3390/jcm12041402/s1; Video S1: The video shows the sliding movement of the pleural line, called “lung sliding” synchronous with the breaths; Video S2. The video shows the so-called “curtain sign” (longitudinal scan at the base of the thorax); during the inspiratory phase, the lung lower segments occupy the costophrenic sinus; this movement is identified on ultrasound by the lung artifacts (A-lines), which descend downwards in the inspiratory phase to cover the liver, while in the expiratory phase, they go back up.

## Abbreviations

ICU	Intensive care unit
PLAPS	Posterolateral and/or pleural alveolar syndrome
COPD	Chronic obstructive pulmonary disease
ARDS	Acute respiratory distress syndrome
CAP	Community-acquired pneumonia
HCAP	Healthcare-associated pneumonia
AIDS	Acquired Immuno Deficiency Syndrome
CEUS	Contrast-enhanced ultrasound
HRCT	High-resolution computed tomography
